# Exploring the synergistic effects of indole acetic acid (IAA) and compost in the phytostabilization of nickel (Ni) in cauliflower rhizosphere

**DOI:** 10.1186/s12870-024-04920-0

**Published:** 2024-04-11

**Authors:** Raheel Khan, Muhammad Junaid Sarwar, Muhammad Shabaan, Hafiz Naeem Asghar, Usman Zulfiqar, Irfan Iftikhar, Nazish Aijaz, Fasih Ullah Haider, Talha Chaudhary, Walid Soufan

**Affiliations:** 1 Institute of Soil and Environmental Sciences, University of Agriculture, Faisalabad, Pakistan; 2 Land Resources Research Institute (LRRI), National Agricultural Research Centre (NARC), Islamabad, Pakistan; 3 Department of Agronomy, Faculty of Agriculture and Environment, The Islamia University of Bahawalpur, Bahawalpur, 63100 Pakistan; 4 School of Biomedical Science, Hunan University, Changsha, Hunan China; 5 MOA Key Laboratory of Soil Microbiology, Rhizobium Research Center, China Agricultural University, Beijing, China; 6 Key Laboratory of Vegetation Restoration and Management of Degraded Ecosystems, South China Botanical Garden, Chinese Academy of Sciences, Guangzhou, 510650 China; 7 University of Chinese Academy of Sciences, Beijing, 100039 China; 8 Faculty of Agricultural and Environmental Sciences, Hungarian University of Agriculture and Life Sciences 2100, Godollo, Hungary; 9 Plant Production Department, College of Food and Agriculture Sciences, King Saud University, Riyadh, 11451 Saudi Arabia

**Keywords:** Antioxidants, Cauliflower, Compost, IAA, Nickel, Phytostabilization

## Abstract

Heavy metals (HMs) contamination, owing to their potential links to various chronic diseases, poses a global threat to agriculture, environment, and human health. Nickel (Ni) is an essential element however, at higher concentration, it is highly phytotoxic, and affects major plant functions. Beneficial roles of plant growth regulators (PGRs) and organic amendments in mitigating the adverse impacts of HM on plant growth has gained the attention of scientific community worldwide. Here, we performed a greenhouse study to investigate the effect of indole-3-acetic acid (IAA @ 10^− 5^ M) and compost (1% w/w) individually and in combination in sustaining cauliflower growth and yield under Ni stress. In our results, combined application proved significantly better than individual applications in alleviating the adverse effects of Ni on cauliflower as it increased various plant attributes such as plant height (49%), root length (76%), curd height and diameter (68 and 134%), leaf area (75%), transpiration rate (36%), stomatal conductance (104%), water use efficiency (143%), flavonoid and phenolic contents (212 and 133%), soluble sugars and protein contents (202 and 199%), SPAD value (78%), chlorophyll ‘a and b’ (219 and 208%), carotenoid (335%), and NPK uptake (191, 79 and 92%) as compared to the control. Co-application of IAA and compost reduced Ni-induced electrolyte leakage (64%) and improved the antioxidant activities, including APX (55%), CAT (30%), SOD (43%), POD (55%), while reducing MDA and H_2_O_2_ contents (77 and 52%) compared to the control. The combined application also reduced Ni uptake in roots, shoots, and curd by 51, 78 and 72% respectively along with an increased relative production index (78%) as compared to the control. Hence, synergistic application of IAA and compost can mitigate Ni induced adverse impacts on cauliflower growth by immobilizing it in the soil.

## Introduction

Intensive industrialization and urbanization have significantly raised heavy metal (HM) contamination in soils, which is a major concern globally [1]. Heavy metals perist in soil for a longer time period and are transferred to human body after their entrance in food chain causing multiple deformities and diseases [2]. Nickel (Ni) is the 24th most abundant element in earth’s crust (3%) after silicon (Si), magnesium (Mg), oxygen (O) and iron (Fe), and ranks as 5th most abundant element on weight basis [3]. However, in excessive concentrations, it exerts adverse impacts on living beings. Natural sources of Ni include volcanic emissions, dust, deforestation, and vegetation whereas, among different anthropogenic sources are crude oil, coal combustion, and ignition of sludge and waste [4]. In environment, excessive use of phosphate fertilizers is also a significant source of Ni contamination [5]. Toxicity limit of Ni in plants ranges from 25 to 50 ppm [6]. Harmful symptoms of Ni toxicity include restricted root and shoot growths, decline in leaf area and chlorosis etc. [7]. During developmental stage, Ni toxicity may result in Fe deficiency, ultimately leading to white banding [8]. Notably, Ni has been found to adversely affect physiological attributes of plants i.e., chlorophyll contents owing to its interference with the uptake of different essential elements [9]. Similarly, Ni exposure has been reported to cause adverse impacts on vegetative and reproductive growth of plants flowering as well as total RNA contents [10]. It is also involved in regulating tumor cell proliferation and metastasis through apoptosis [11]. Furthermore, Ni exposure may result in lichenification, eczema and erythema on skin upon exposure, and may even cause skin cancer and allergies [12].

Remediation of HM contaminated soils is imperative to fulfill the dietary demands of ever growing global population, which has resulted in the shrinkage of agricultural lands [13, 14]. Numerous physiochemical and biological methods can be used to eliminate HM contamination but their associated costs and complexity limit their adoption on larger scales [15, 16]. Furthermore, these techniques only alter the nature of pollutants, and do not eliminate them from system. Biological approaches, on the other hand, are cost-effective, environment friendly, and have a high public acceptance [17, 18]. Phytoremediation involving the use of green plants, is a sustainable alternative for the removal of hazardous metals. It is an efficient and inexpensive technique as compared to other conventional methods and can be applied to eliminate HM as well as organic pollutants from soil [19].

Provision of plant growth regulators (PGRs) under HM stress can enhance plant tolerance to these toxic contaminants [20]. They are naturally occurring organic compounds that promote plant biomass, yield, and root growth, as well as regulate antioxidative systems under normal and contaminated conditions [21]. Auxin (IAA), one of the most important natural plant hormones, is commercially used to enhance crop yield, and regulate plant growth under stressed soil conditions [22]. Auxin sustains plant growth and development chiefly by its biosynthesis, transport as well as signal transduction, and is generally referred to as growth hormone due to its involvement in almost every growth and developmental process [23]. Exogenous application of auxin (IAA) can overcome the hazardous effects of HMs on plant growth, while addition of a suitable amount of IAA under metal-contaminated soil can improve plant growth, biomass, and yield [24].

Organic amendments play keen roles in reducing HM toxicity to plants [25]. Among different organic amendments, application of compost has been found to be effective in reducing the bioavailability of HMs by their immobilization owing to different functional groups such as hydroxyl (OH^−^) or carboxylic acids (COOH^−^) [26]. Addition of compost as an organic fertilizer can also lower metal solubility by forming stable chelates and reducing its bioavailable fractions in soil solution [27]. Furthermore, combined application of PGRs and compost has the potential to improve plant growth under HM-stress [28, 29]. Efficiency of phytoremediation processes in improving the plant growth under stressed conditions has been reported to be affected by different factors and multiple researchers have tried to improve its efficiency by combining with different organic amendments. However, fewer reports exist in literature regarding the combination of organic amendments alongside different PGRs for sustaining plant growth under heavy metal stressed conditions. Hence, we hypothesized that combined application of IAA and compost can significantly alleviate the growth and survival of cauliflower under Ni stress by immobilizing it in soil. Based on the hypothesis, the objectives of present study were to investigate the potential effects of auxin (IAA) and compost in optimizing the growth, yield and physiology of cauliflower under Ni-contaminated soil conditions.

## Materials and methods

A greenhouse study was carried out at the wire house located at the Institute of Soil and Environmental Sciences (ISES), University of Agriculture Faisalabad (31.26 °N, 73.06 °E) for evaluating the effectiveness of plant growth regulator i.e., ‘IAA’ in combination with compost in reducing nickel (Ni) uptake and enhancing cauliflower growth under Ni stress. Soil was contaminated two weeks prior to the experiment with nickel sulfate (NiSO_4_) salt at 60 mg kg^− 1^. Similarly, compost was amended @ 1% w/w prior to seed sowing. Solution of IAA (10^− 5^ M) was prepared for cauliflower seed priming as per treatment plan. Physicochemical attributes of soil and compost used in the current experiment have been elaborated in Table 1. Seeds of cauliflower (Hybrid FD-I) were taken from Vegetable Research Institute, Faisalabad, Pakistan. Each pot was filled with 7 kg of uniformly mixed and air-dried soil. Pots were arranged by following the completely randomized design (CRD) having three replicates each under 3-factor factorial arrangement. Initially, 5 seeds of cauliflower per pot were sown, and 10 days after germination, the seedlings were thinned down to 2 per pot. The experiment lasted for 120 days during which all the essential agronomic practices were followed, and the recommended NPK (100, 60, and 60 kg ha^− 1^) requirements were met by applying urea, diammonium phosphate, and muriate of potash at the recommended doses. As the plants reached maturity, a series of biochemical, physiological, and antioxidant-related analyses were conducted. Growth and yield parameters were assessed at the time of harvest.

**Table 1:** Physicochemical attributes of compost and soil used in the experiment

Properties	Unit	Soil	Compost
Organic matter	%	0.61	4.58
pH	-	7.4	8.22
Electrical Conductivity	dS m^− 1^	1.56	2.19
Total Nitrogen	%		1.35
Available Phosphorous	mg kg^− 1^	6.78	6.45
Extractable Potassium	mg kg^− 1^	165.7	91.5
Cation Exchange Capacity	cmolc kg^− 1^	1.44	
Volumetric moisture contents	%	35.98	
Nickel concentration	mg kg^− 1^	Non-detectable	Non-detectable

### Chlorophyll contents

To determine the chlorophyll contents, method outlined by Arnon [30] was followed, and process included extracting 100 mg of fresh foliage sample with 8 mL of 80% (v/v) acetone in a pre-chilled mortar, followed by filtering the extract mixture and adjusting the volume to 10 mL with cold acetone. The optical density of the solution was measured using a UV-Vis Spectrophotometer (ANA-720 W, Tokyo Photoelectric Company Limited, Japan) at wavelengths of 663 nm, 645 nm, and 480 nm respectively. The chlorophyll pigments were quantified using a specific formula and expressed as mg g^− 1^ FW.

$$\begin{array}{l}\text{C}\text{h}\text{l}\text{o}\text{r}\text{o}\text{p}\text{h}\text{y}\text{l}\text{l} {\prime }\text{a}{\prime }\\=\left[12.7 \left(663 nm OD\right)-2.69 \left(645 nm OD\right)\times \frac{sample\,volume}{1000} \times fresh\,weight\right.\end{array}$$

$$\begin{array}{l} \text{C}\text{h}\text{l}\text{o}\text{r}\text{o}\text{p}\text{h}\text{y}\text{l}\text{l} {\prime }\text{b}{\prime }\\=\left[[22.9 \left(645 nm OD\right)-4.68 (663 nm OD)\times \frac{sample\,volume}{1000} \times fresh\,weight\right.\end{array}$$

$$\begin{array}{l} \text{C}\text{a}\text{r}\text{o}\text{t}\text{e}\text{n}\text{o}\text{i}\text{d}\,\text{c}\text{o}\text{n}\text{t}\text{e}\text{n}\text{t}\text{s} =\\\left[\frac{OD at 480 nm + 0.114 \left(OD at 663 nm\right)-0.638 \left(OD at 645 nm\right)}{2500}\right] \times 100\end{array}$$

Similarly, for measuring the relative chlorophyll contents in terms of SPAD value, chlorophyll meter (SPAD-502) was used at 10:00 a.m.

### Gas exchange

The determination of gas exchange attributes including stomatal and sub-stomatal conductance, vapor pressure deficit (VPD), water use efficiency (WUE), transpiration, and photosynthetic rates, was carried out by using the Combined Infrared Analyzing System (CIRAS-3). These measurements were conducted between 8 and 10 a.m., and mean values were calculated from fully expanded top leaves (three leaves per experimental unit) under a photosynthetic photon flux density of 1400 µmol m^− 2^ s^− 1^.

### Antioxidants measurements

Among different antioxidants, catalase (CAT) activities were determined based on H_2_O_2_ decomposition at 240 nm in 3 mL of reaction mixture [31]. Similarly, modified NBT method was used for determination of superoxide dismutase (SOD) activity [32]. Peroxidase (POX) and ascorbate peroxidase (APX) activities were assayed by following the methods of Zhang and Kirkham [33] and Nakano and Asada [34]. During measurement of POX activities, absorbance of reaction mixture was checked at 470 nm. In contrast, APX activities were based on the reduction in absorbance of reaction mixture during oxidation of ascorbate.

### Malondialdehyde (MDA) contents and electrolyte leakage (EL)

Malondialdehyde (MDA) contents were determined using the method outlined by Heath and Packer [35], which involved reaction of thio-barbituric acid (TBA) with tricarboxylic acid (TCA) followed by measurement of their absorbance at 532 and 450 nm. Similarly, electrolyte leakage (EL) was determined by immersing and incubating cauliflower leaves in distilled water for one day and measuring the EC of that water (labelled as EC1) by EC meter (Ohaus conductivity meter model Starter 3100 C). Later, this solution was autoclaved at 121 °C for 20 min to kill all the tissues and release all electrolytes. Subsequently, the solution was cooled down, and again EC was measured (labelled as EC2). The following formula was used to measure electrolyte leakage [36].

$$\text{E}\text{l}\text{e}\text{c}\text{t}\text{r}\text{o}\text{l}\text{y}\text{t}\text{e}\,\text{l}\text{e}\text{a}\text{k}\text{a}\text{g}\text{e} \left(\text{\%}\right) =\frac{EC1}{EC2} \times 100$$

### Proline determination

Proline contents were measured by using the method of Bates et al. [37], which involved homogenization of leaves in salicylic acid. The concentration of H_2_O_2_ in leaves as oxidative stress marker, was determined based on homogenization of 50 mg fresh tissue in TCA (0.1%) and its subsequent centrifugation [38].

### Nutrient uptake

Determination of nutrient uptake in plant samples was done by digestion process. For the digestion, the method of Wolf [39] was used, and volume of digested material was made up to the mark by distilled water. Later, this material was stored for nutrient analyses. For determination of nitrogen (N) and phosphorous (P), Kjeldahl distillation apparatus and Spectrophotometer were used. Whereas, for potassium (K), calcium (Ca) and magnesium (Mg), Flame photometer (Jenway PFP-7) was used.

### Biochemical analyses

To determine the total soluble sugar (TSS), the anthrone sulfuric acid method was employed. The absorbance of the reaction mixture was measured at 625 nm using a Spectrophotometer (ANA-720 W, Tokyo Photoelectric Company Limited, Japan) [40]. For free amino acid analysis, the ninhydrin method developed by Lee and Takahashi [41] was utilized, with absorbance measured at 570 nm. Total protein content was determined by assessing the binding of protein with brilliant blue G-250, with absorbance increasing and results compared to a standard curve [42]. For phenolic determination, leaves were homogenized in ethanol, filtered, and the supernatant was used for phenolic content determination using gallic acid as a standard [43]. The determination of flavonoid contents in the leaves involved an aluminum chloride calorimetric method with quercetin as the standard [44].

### Determination of total metal concentration

Mixture was digested in a solution consisting of HNO_3_: H_2_SO_4_: HClO_4_ at a ratio of 5:2:1 and heated up to 80 °C until a clear solution was obtained. The solution was filtered by using Whatman filter # 42, and resulting mixture was further diluted up to 50 mL. The concentration of Ni in soil and plants samples was analyzed using an Atomic Absorption Spectrophotometer (Thermo Electron S series). Similarly, bioconcentration (BCF) and translocation factors (TF) were calculated by following the method of Ali et al. [45].

### Relative production index (RPI)

It quantified the effect of HMs stress on variations in dry matter production and was computed using the following formula [46].

$$\text{R}\text{P}\text{I} =\frac{\text{D}\text{r}\text{y}\,\text{m}\text{a}\text{t}\text{t}\text{e}\text{r}\,\text{p}\text{r}\text{o}\text{d}\text{u}\text{c}\text{e}\text{d}\,\text{u}\text{n}\text{d}\text{e}\text{r}\,\text{N}\text{i}\,\text{s}\text{t}\text{r}\text{e}\text{s}\text{s}}{\text{D}\text{r}\text{y}\,\text{m}\text{a}\text{t}\text{t}\text{e}\text{r}\,\text{p}\text{r}\text{o}\text{d}\text{u}\text{c}\text{e}\text{d}\,\text{u}\text{n}\text{d}\text{e}\text{r}\,\text{n}\text{o}\text{r}\text{m}\text{a}\text{l}\,\text{c}\text{o}\text{n}\text{d}\text{i}\text{t}\text{i}\text{o}\text{n}\text{s}}$$

### Statistical analysis

The data obtained for each parameter were analyzed using a 3-way analysis of variance (ANOVA) with “Statistix 8.1” software. In addition, a multiple mean-wise comparison test was conducted using Tukey’s Honestly Significant Difference (HSD) test at a significance level of *p* ≤ 0.05. Means and standard errors were calculated by using Microsoft Excel. Moreover, a Pearson Correlation analysis between Ni uptake and various plant attributes was carried out in RStudio. Based on the results, a correlogram was generated using the ‘cor’ function and the publicly available ‘corrplot’ package [47].

## Results

### Growth and yield attributes

**Fig. 1:**
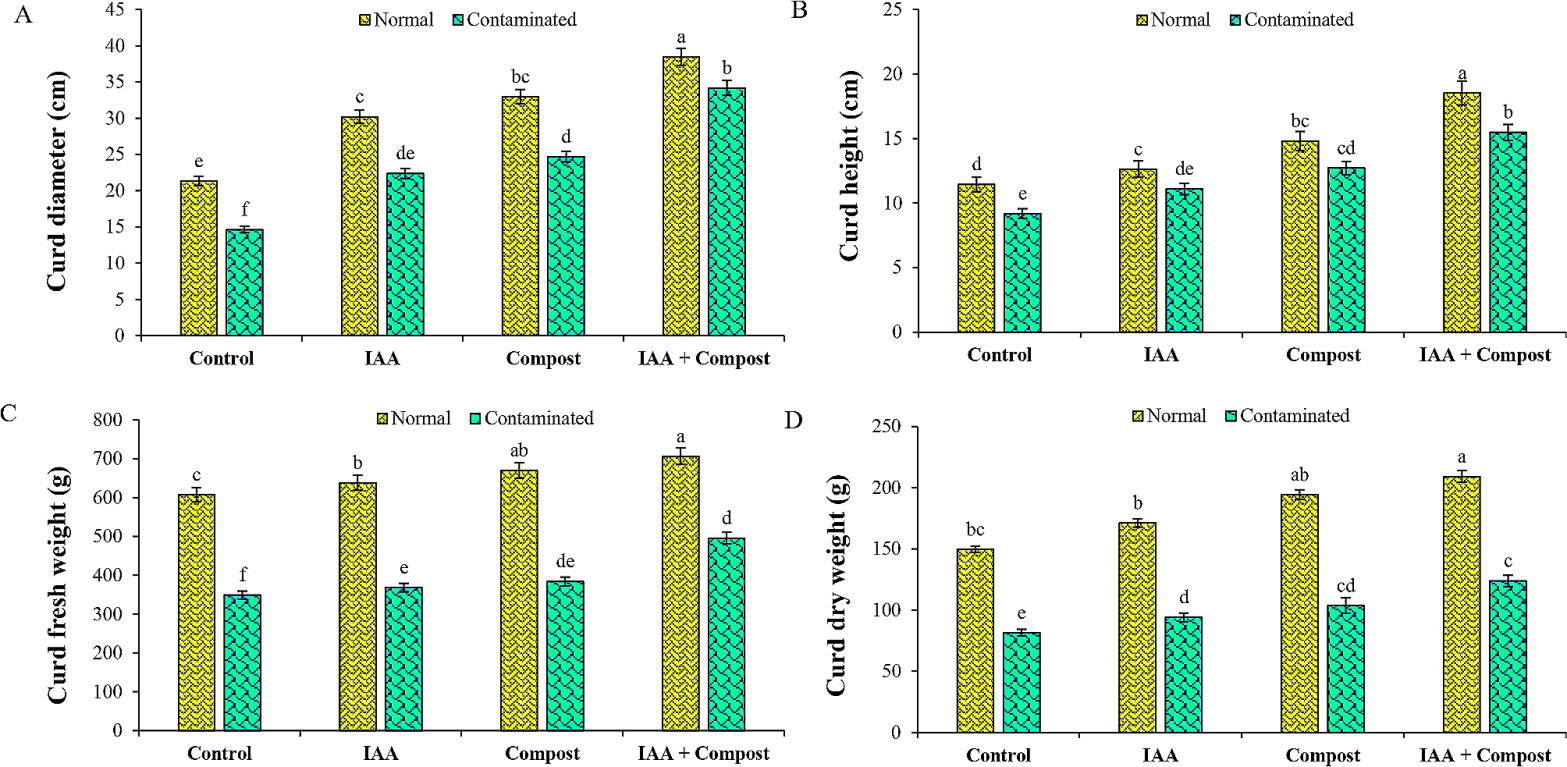
Effect of individual and combined application of IAA and compost on yield attributes of cauliflower i.e., (**A**) Curd diameter (cm), (**B**) Curd height (cm), (**C**) Curd fresh weight (g) and (**D**) Curd dry weight (g) under Ni stress. Bars displaying same alphabets are statistically non-significant at *P* < 0.05

Data regarding various growth (Table 2) and yield (Fig. 1) attributes revealed that among different treatments, sole application of compost and IAA increased shoot length (23 and 13%), root length (63 and 44%), shoot fresh weight (12 and 6%), root fresh weight (34 and 15%), shoot dry weight (34 and 15%), root dry weight (100 and 67%), leaf area (46 and 39%), number of leaves per plant (100 and 77%), curd fresh weight (10 and 6%), curd dry weight (27 and 15%), curd diameter (125 and 106%) and curd height (49 and 36%) as compared to control under Ni stress. However, combined application of PGR and compost under Ni stress performed comparatively better and increased shoot and root lengths (49 and 76%), shoot fresh and dry weights (24 and 136%), root fresh and dry weights (47 and 140%), fresh and dry weights of cauliflower curd (18 and 52%), curd height and diameter (38 and 134%), as well as leaf area and number of leaves per plant (75 and 215%) as compared to control treatment.

**Table 2:** Effect of sole and combined application of IAA and compost on growth attributes of cauliflower under Ni stress

**Treatments**	**Shoot length (cm)**		**Root length (cm)**		**Shoot fresh weight (g)**		**Shoot dry weight (g)**	
	**Normal**	**Contaminated**	**Normal**	**Contaminated**	**Normal**	**Contaminated**	**Normal**	**Contaminated**
**Control** **IAA** **Compost** **IAA + Compost**	24.14 ± 1.85 d 27.13 ± 2.04 c 28.31 ± 2.01 bc 31.32 ± 1.99 a	19.77 ± 1.13 f 22.39 ± 2.04 e 24.31 ± 1.98 d 29.47 ± 2.19 b	15.09 ± 1.02 bc 16.61 ± 1.39 b 17.37 ± 2.04 ab 18.97 ± 1.36 a	9.17 ± 1.01 d 13.38 ± 0.98 c 14.91 ± 1.12 bc 16.13 ± 1.38 b	449 ± 0.57 d 469.16 ± 0.59 c 483.5 ± 0.86 b 509 ± 0.57 a	258.5 ± 0.86 h 274 ± 0.57 g 288.7 ± 0.72 f 320.52 ± 0.74 e	70 ± 0.28 d 84.54 ± 0.29 c 94 ± 0.57 b 108.83 ± 0.59 a	29.16 ± 0.43 g 43.5 ± 0.76 f 54 ± 0.57 e 68.8 ± 0.59 d
	**Root fresh weight (g)**		**Root dry weight (g)**		**Leaf area (cm** ^**2**^ **)**		**No. of leaves plant** ^**− 1**^	
	**Normal**	**Contaminated**	**Normal**	**Contaminated**	**Normal**	**Contaminated**	**Normal**	**Contaminated**
**Control** **IAA** **Compost** **IAA + Compost**	49 ± 0.57 d 57 ± 0.57 c 64.83 ± 0.43 b 76.16 ± 0.72 a	29 ± 0.57 h 33.3 ± 0.43 g 39 ± 0.28 f 42.5 ± 0.28 e	20.5 ± 0.40 d 25 ± 0.81 c 28.75 ± 0.20 b 33.75 ± 1.42 a	8.24 ± 0.21 g 13.75 ± 0.60 f 16.5 ± 0.40 e 19.75 ± 0.60 d	174.61 ± 4.4 bc 180.97 ± 2.91 b 185.36 ± 3.75 b 230.41 ± 2.19 a	119.47 ± 3.15 d 166.45 ± 2.47 c 174.19 ± 4.71bc 209.32 ± 2.25 ab	8.23 ± 0.98 cd 9.32 ± 0.74 c 10.11 ± 1.07 ab 11.34 ± 1.24 a	3.19 ± 0.08 f 5.65 ± 0.10 e 6.39 ± 0.04 d 10.05 ± 0.09 b

**Abbreviations: IAA = Indole-3-acetic acid**

**Means sharing same alphabets are statistically non-significant at***P* < 0.05

### Gas exchange attributes

**Fig. 2:**
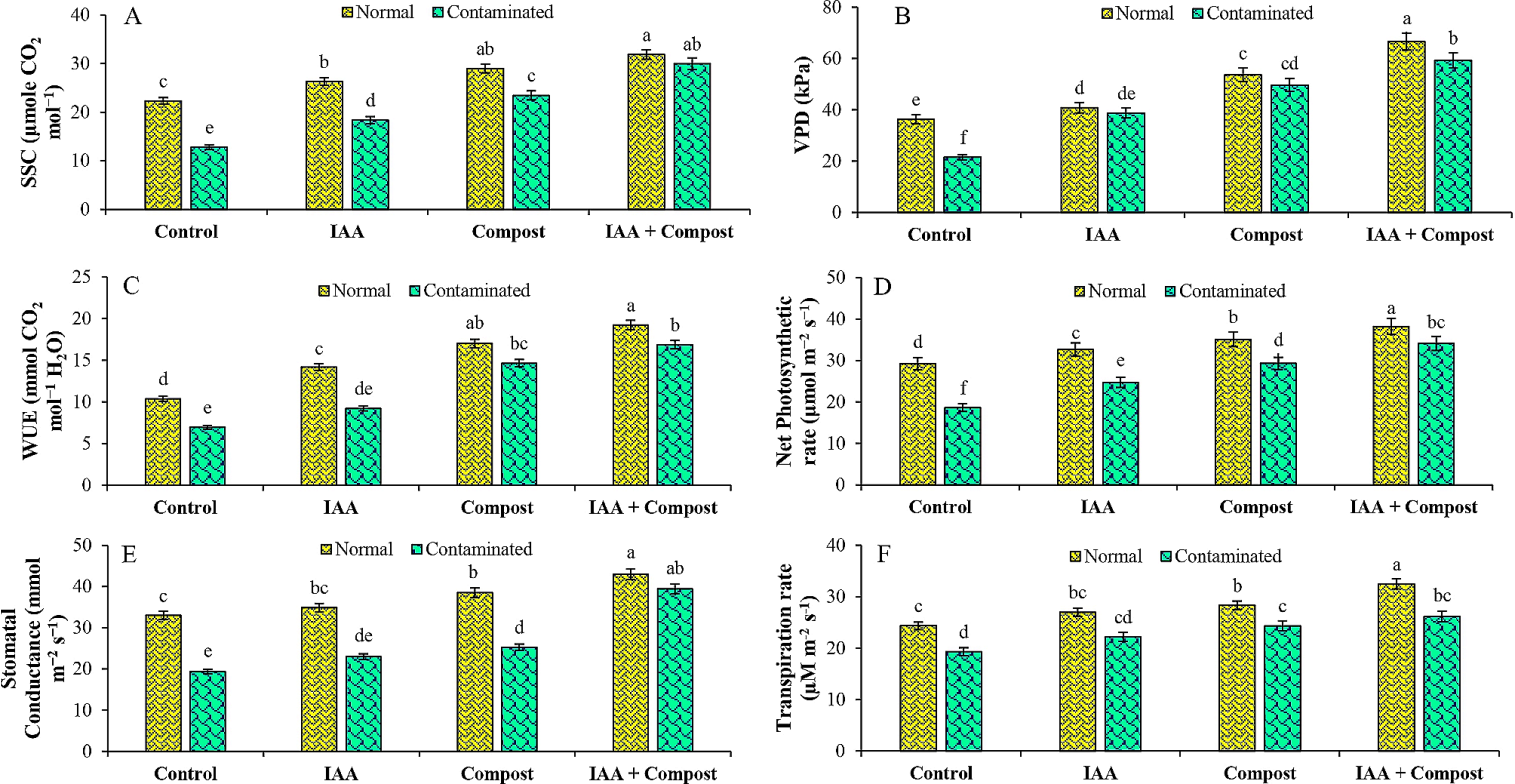
Effect of individual and combined application of IAA and compost on gas exchange attributes i.e., (**A**) Sub-stomatal conductance (SSC; µmole CO_2_ mol^− 1^), (**B**) Vapor pressure deficit (VPD; kPa), (**C**) Water use efficiency (WUE; mmol CO_2_ mol^− 1^ H_2_O), (**D**) Net photosynthetic rate (µmol m^− 2^ s^− 1^), (**E**) Stomatal Conductance (mmol m^− 2^ s^− 1^), and (**F**) Transpiration rate (TR; µM m^− 2^ s^− 1^) under Ni stress. Bars displaying same alphabets are statistically non-significant at *P* < 0.05

In current study, data regarding the role of IAA and compost on various gas exchange attributes (Fig. 2) exhibited a negative correlation between these traits and Ni stress, which was significantly ameliorated by combined and individual applications of IAA and compost. In individual application of IAA and compost, significant increments in transpiration rate (15 and 26%), stomatal conductance (19 and 31%), net photosynthetic rate (33 and 57%), water use efficiency (32 and 111%), sub-stomatal conductance (43 and 83%) and vapor pressure deficit (81 and 131%) were pragmatic. Whereas these results were augmented under the combined application of IAA and compost, that increased these gas exchange attributes i.e., transpiration rate (35%), stomatal conductance (104%), net photosynthetic rate (83%), water use efficiency (143%), sub-stomatal conductance (134%) and vapor pressure deficit (176%) as compared to control treatment under Ni contamination (Fig. 2).

### Antioxidant production

**Fig. 3:**
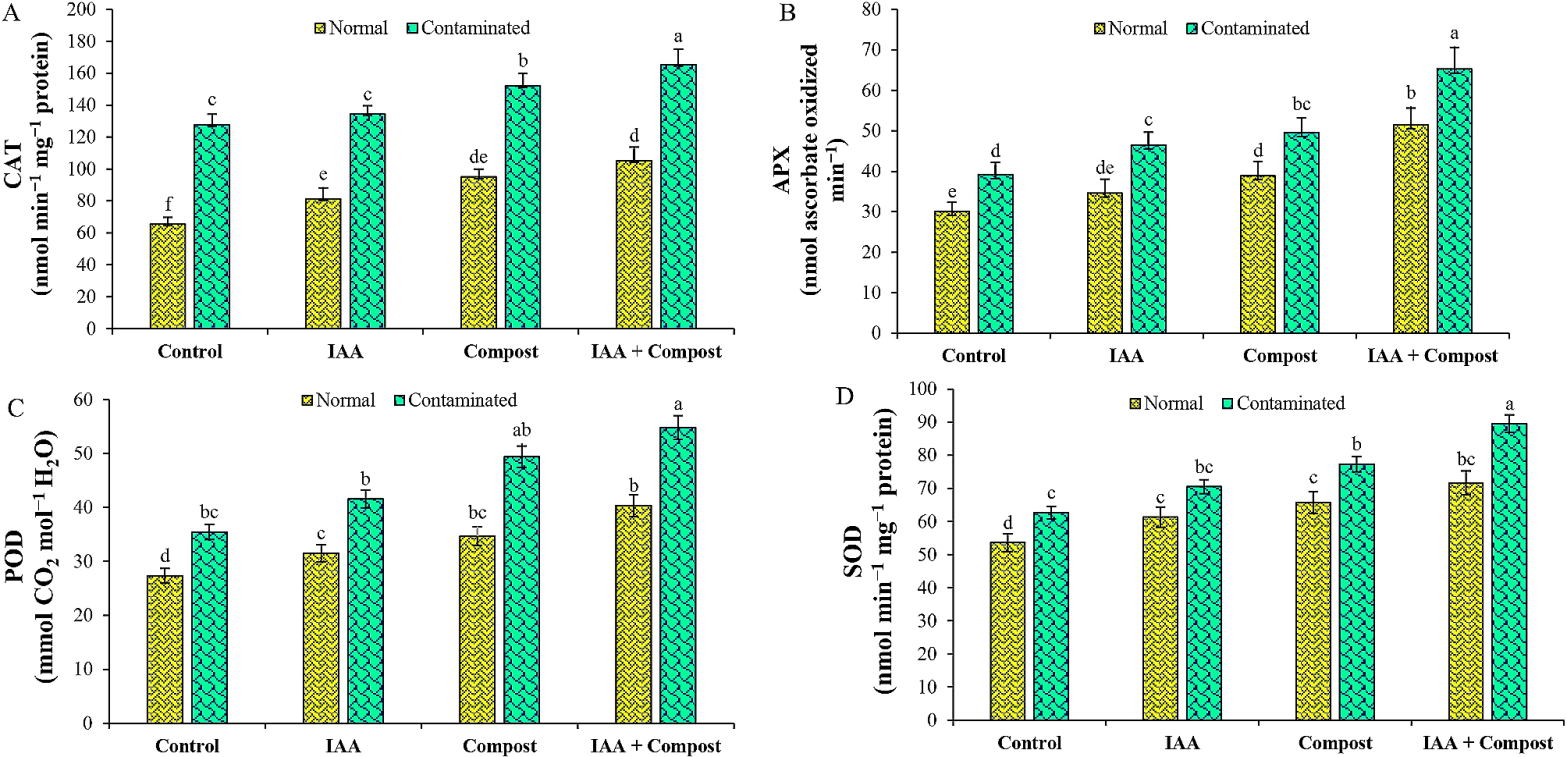
Effect of individual and combined application of IAA and compost on antioxidant activities i.e., (**A**) Catalase (CAT; nmol min^− 1^ mg^− 1^ protein), (**B**) Ascorbate peroxidase (APX; nmol ascorbate oxidized min^− 1^), (**C**) Peroxidase (POD; mmol CO_2_ mol^− 1^ H_2_O), and (**D**) Superoxide dismutase (SOD; nmol min^− 1^ mg^− 1^ protein) under Ni stress. Bars displaying same alphabets are statistically non-significant at *P* < 0.05

Data in terms of antioxidant production (Fig. 3) depicted that exposure to Ni stress significantly enhanced the level of antioxidant production in cauliflower leaves. However, sole application of IAA and compost decreased the activities of different antioxidants such as catalase (13 and 20%), superoxide dismutase (5 and 9%), ascorbate peroxidase (45 and 48%) and peroxidase (34 and 44%) as compared to relevant control conditions under Ni stress. Contrastingly, IAA application in association with compost led to a further decline in these antioxidant activities, and decreased CAT, SOD, APX and POD activities by 27, 17, 56 and 55% respectively, under Ni stress, as compared to relevant control conditions.

### Electrolyte leakage (EL) and membrane permeability

**Fig. 4:**
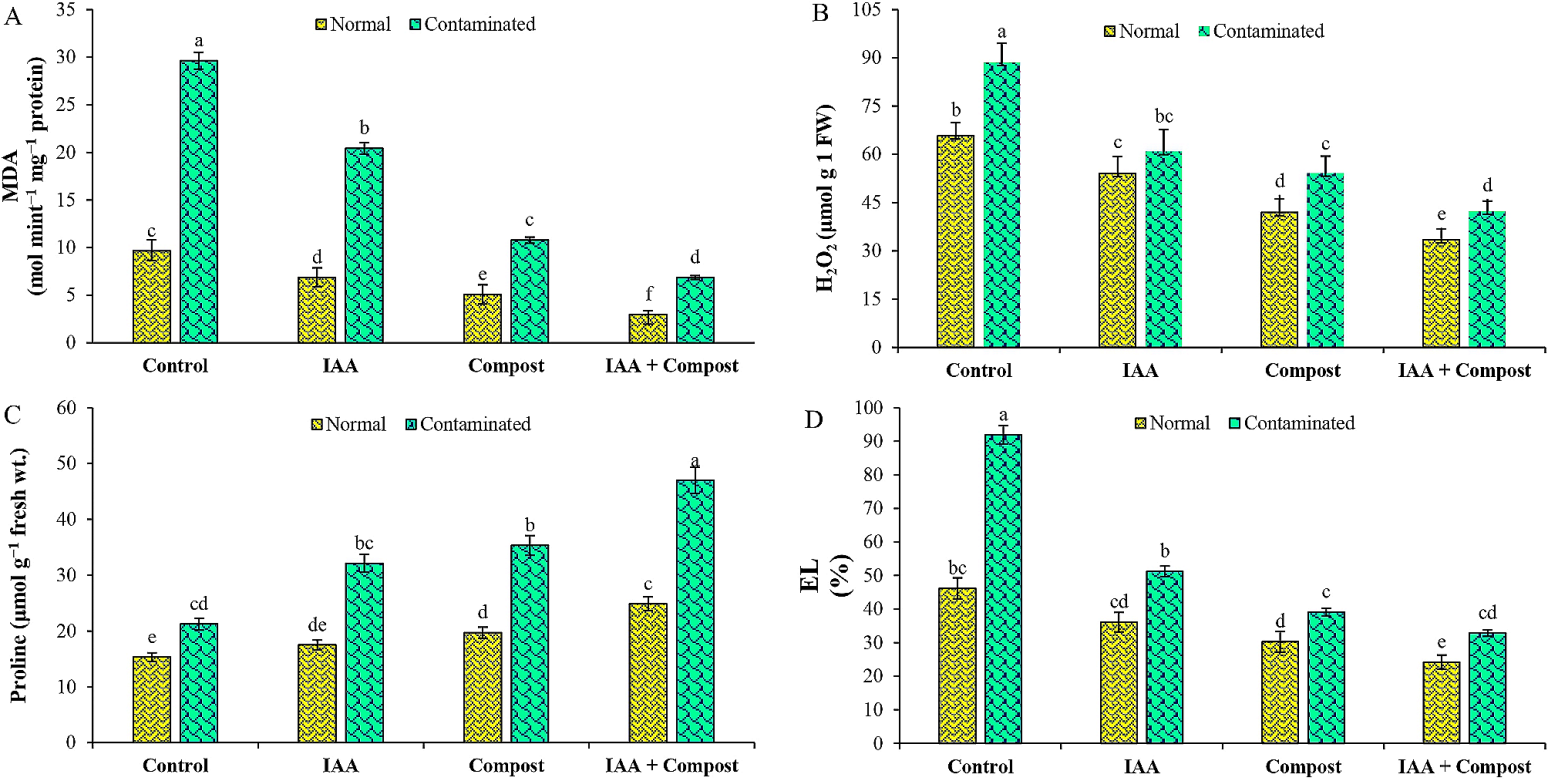
Effect of individual and combined application of IAA and compost on (**A**) Malondialdehyde contents (MDA; n mol mint^− 1^ mg^− 1^ protein), (**B**) Hydrogen peroxide (H_2_O_2_; µmol g^− 1^ FW), (**C**) Proline contents (µmol g^− 1^ fresh weight), (**D**) Electrolyte Leakage (EL; %) under Ni stress. Bars displaying same alphabets are statistically non-significant at *P* < 0.05

We observed a strong positive correlation between Ni stress and various attributes such as electrolyte leakage, malondialdehyde (MDA) contents and H_2_O_2_ production (Fig. 4). These adverse impacts on membrane functions were significantly restored by individual and co-application of IAA and compost. We observed that Ni stress significantly enhanced the EL, MDA and H_2_O_2_ by 99, 414 and 35% respectively as compared to control treatment. However, application of compost and IAA decreased the MDA contents (39 and 19%), EL (57 and 44%) and H_2_O_2_ (39 and 31%) production as compared to respective control treatment. Combined application of IAA and compost further decreased the MDA and H_2_O_2_ contents along with EL by 62, 52 and 64% respectively, under Ni stress. Furthermore, IAA and compost as well as their integrated applications enhanced the proline production in cauliflower leaves by 48, 99 and 149% respectively, as compared to control treatment (Fig. 4).

### Chlorophyll contents

**Fig. 5:**
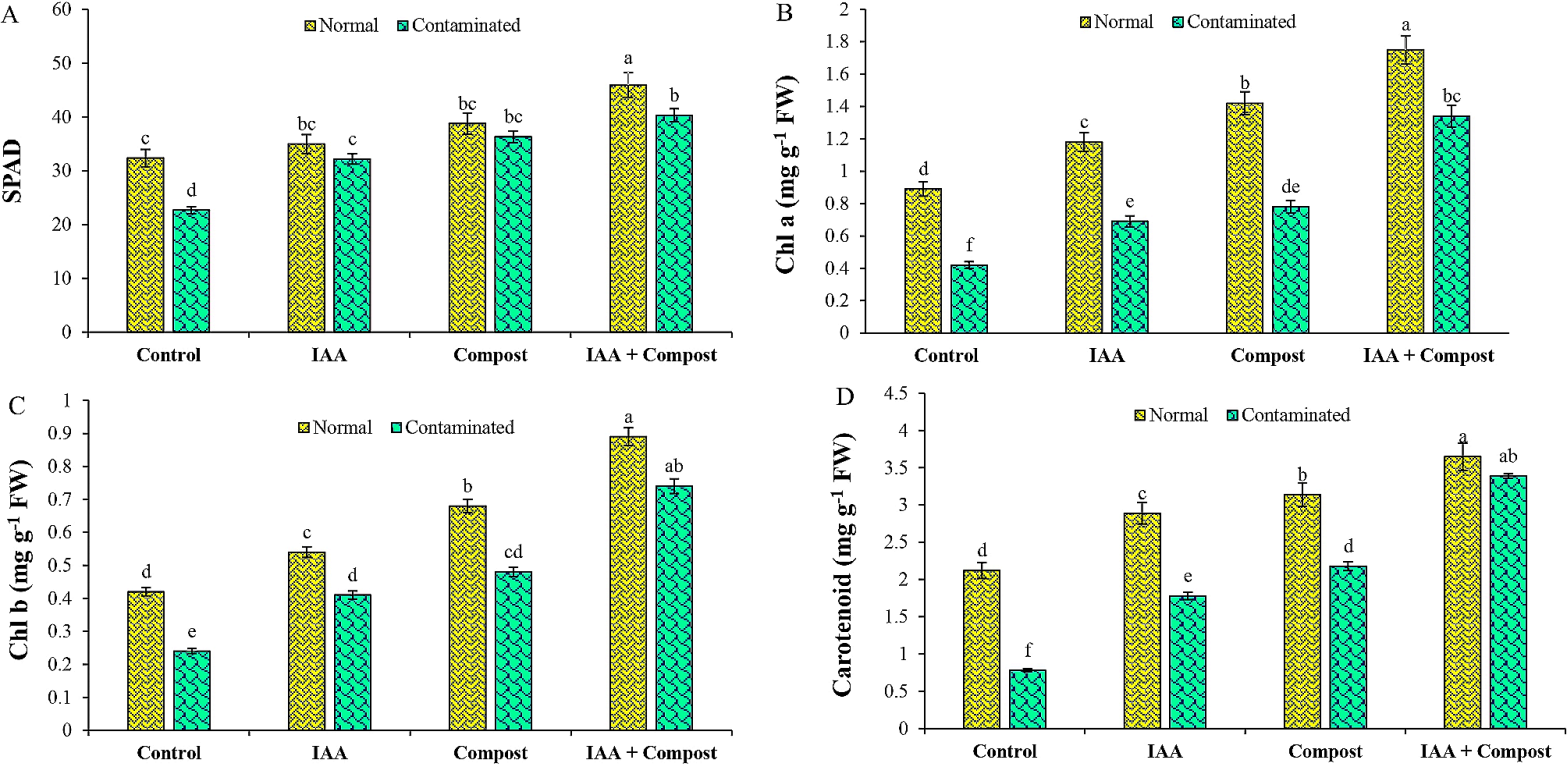
Effect of individual and combined application of IAA and compost on chlorophyll contents (**A**) SPAD value, (**B**) chlorophyll ‘a’ (mg g^− 1^ FW), (**C**) chlorophyll ‘b’ (mg g^− 1^ FW), and (**D**) Carotenoid contents (mg g^− 1^ FW) under Ni stress. Bars displaying same alphabets are statistically non-significant at *P* < 0.05

Results regarding chlorophyll contents such as SPAD value, chlorophyll ‘a’, ‘b’ and carotenoid contents (Fig. 5) revealed a significant negative correlation with Ni contamination. Exposure to Ni stress led to a significant decline in SPAD value, chlorophyll ‘a’, ‘b’ and carotenoid contents by 30, 53, 43 and 63% respectively, as compared to control treatment. However, we observed that application of IAA, compost and their joint application enhanced the SPAD value (42, 60 and 78%), chlorophyll ‘a’ (64, 86 and 219%), chlorophyll ‘b’ (71, 100 and 208%) and carotenoid contents (128, 179 and 334%) as compared to respective control treatment under Ni contamination.

### Nutrient uptake

**Fig. 6:**
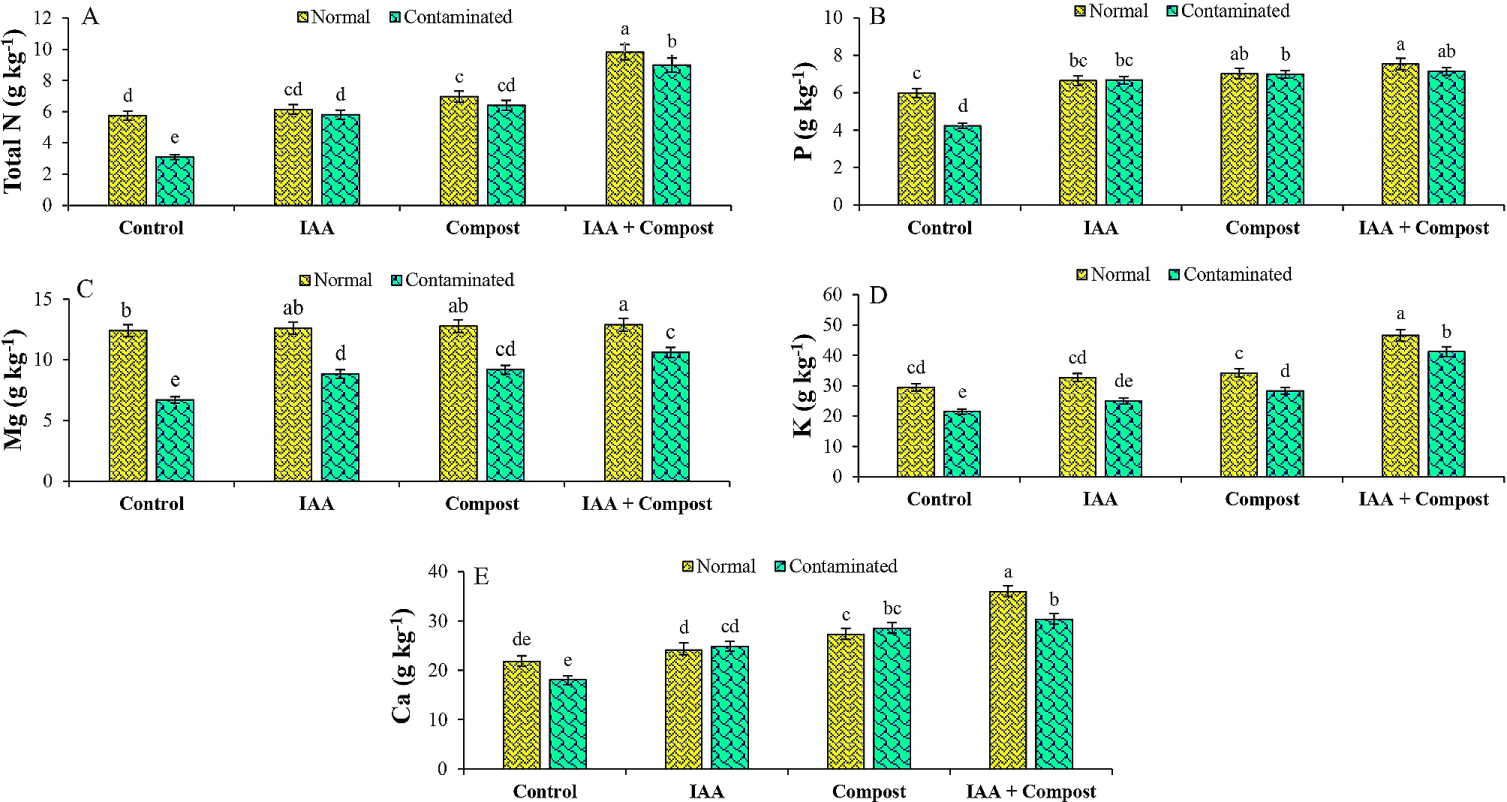
Effect of individual and combined application of IAA and compost on nutrient uptake by plant A) Nitrogen (N; g kg^− 1^, B) Phosphorous (P; g kg^− 1^), C) Magnesium (Mg; g kg^− 1^), D) Potassium (K; g kg^− 1^) and E) Calcium (Ca; g kg^− 1^) under Ni stress. Bars displaying same alphabets are statistically non-significant at *P* < 0.05

Upon exposure to Ni stress, uptake of essential nutrients was significantly decreased in plant, leading to severe reduction in its growth, which was significantly counteracted by compost and IAA applications (Fig. 6). Uptake of N, P, K, Ca, and Mg was reduced by 46, 29, 27, 17 and 29% respectively, as compared to control treatment. These negative impacts on nutrient uptake were significantly mitigated by compost and IAA, as they increased the N (107 and 88%), P (65 and 58%), K (32 and 16%), Ca (58 and 38%) and Mg (37 and 32%) as compared to control treatment. Moreover, combined application of IAA and compost significantly improved N (191%), P (69%), K (92%), Ca (68%) and Mg (59%) under Ni contaminated soil conditions.

### Osmoprotectants production

**Fig. 7:**
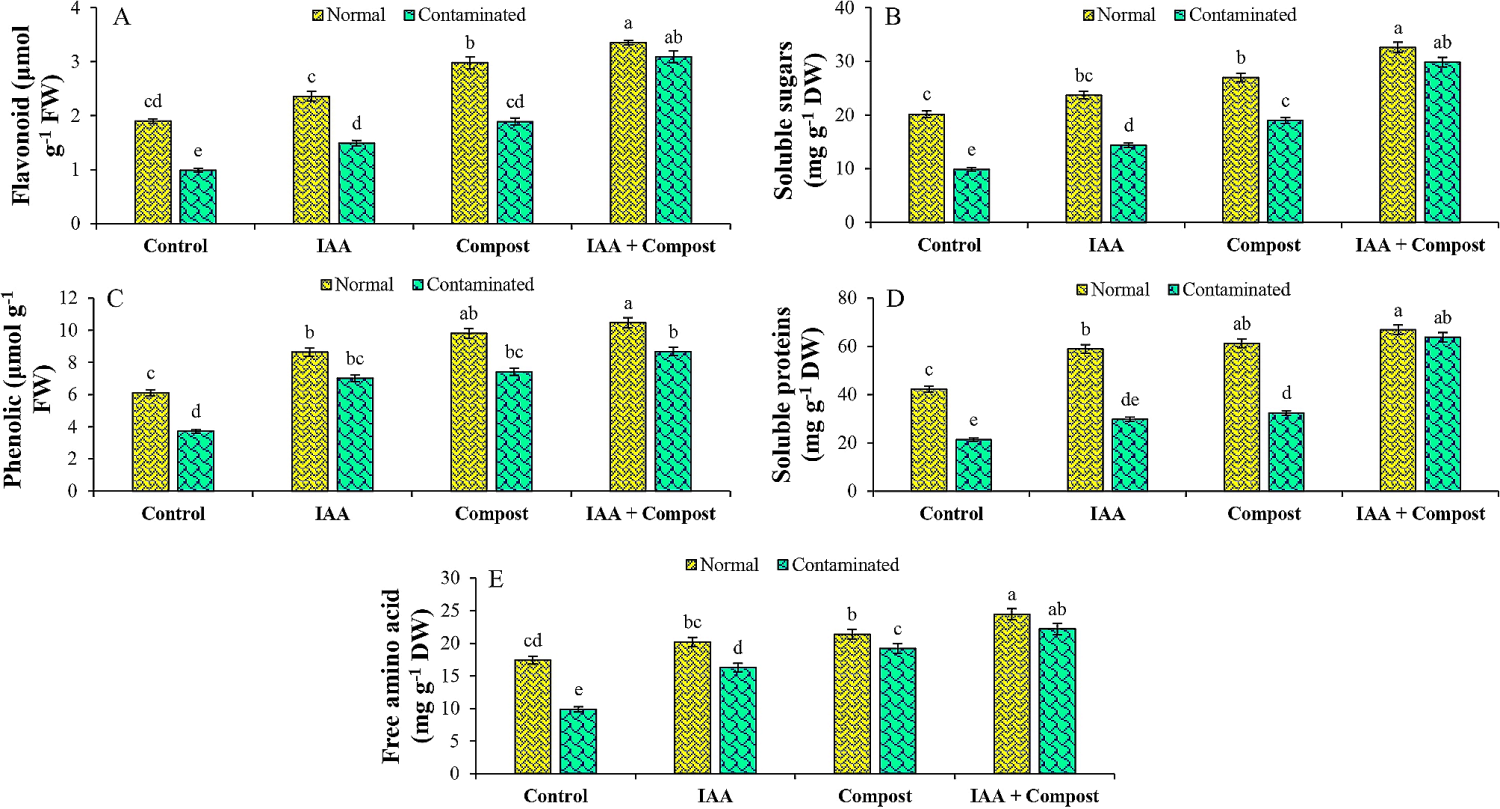
Effect of individual and combined application of IAA and compost on osmoprotectants production (**A**) Flavonoid contents (µmol g^− 1^ FW), (**B**) Soluble sugars (mg g^− 1^ DW), (**C**) Phenolic contents (µmol g^− 1^ FW), (**D**) Soluble proteins (mg g^− 1^ DW) and (**E**) free amino acids (mg g^− 1^ DW) under Ni stress. Bars displaying same alphabets are statistically non-significant at *P* < 0.05

In current experiment, exposure to Ni stress significantly reduced the production of different osmoprotectants such as flavonoid contents, soluble sugars and proteins, phenolic contents, and total free amino acids by 48, 51, 50, 39 and 43% respectively, as compared to control treatment (Fig. 7). However, production of these osmolytes was significantly enhanced by individual and combined application of IAA and compost. In terms of individual application, flavonoid contents (91 and 51%), soluble sugars (92 and 45%), soluble proteins (52 and 39%), phenolic contents (88 and 99%), and total free amino acids (95 and 65%) were considerably enhanced by compost and IAA applications respectively. Integrated application of IAA and compost further enhanced these attributes as compared to individual application and improved flavonoid contents (212%), soluble sugars (202%), soluble proteins (133%), phenolic contents (199%), and total free amino acids (125%), in comparison with their relevant control treatment, under Ni stress.

### Nickel (Ni) concentration

**Fig. 8:**
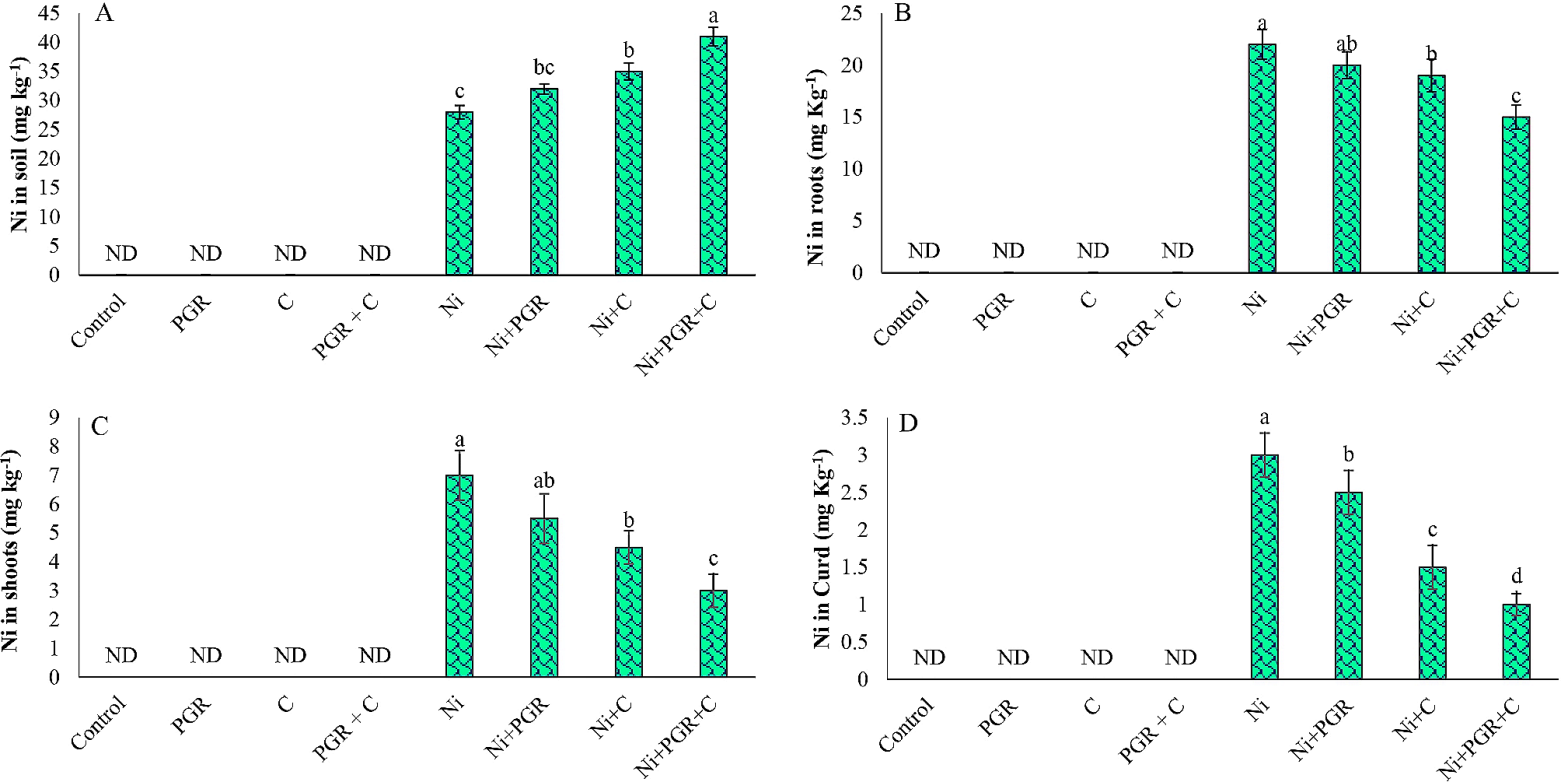
Effect of individual and combined application of IAA and compost on (**A**) leftover Ni concentration in soil (mg kg^− 1^), (**B**) Ni concentration in roots (mg kg^− 1^), (**C**) Ni concentration in shoots (mg kg^− 1^) and (**D**) Ni concentration in curd (mg kg^− 1^). Bars displaying same alphabets are statistically non-significant at *P* < 0.05

Application of compost and IAA alone and in combination suppressed Ni uptake in different plant parts by immobilizing it in soil (Fig. 8). We observed that individual soil supplementation with IAA and compost reduced Ni concentration in roots (14 and 9%), shoots (36 and 21%) and curd (50 and 17%) of cauliflower along with residual soil Ni concentration (25 and 14%), as compared to relevant control. Similarly, combined application of IAA and compost further decreased Ni concentration in root, shoot and curd of cauliflower by 32, 57 and 67% respectively, in addition to residual soil Ni concentration (46%) as compared to corresponding control treatment.

**Fig. 9:**
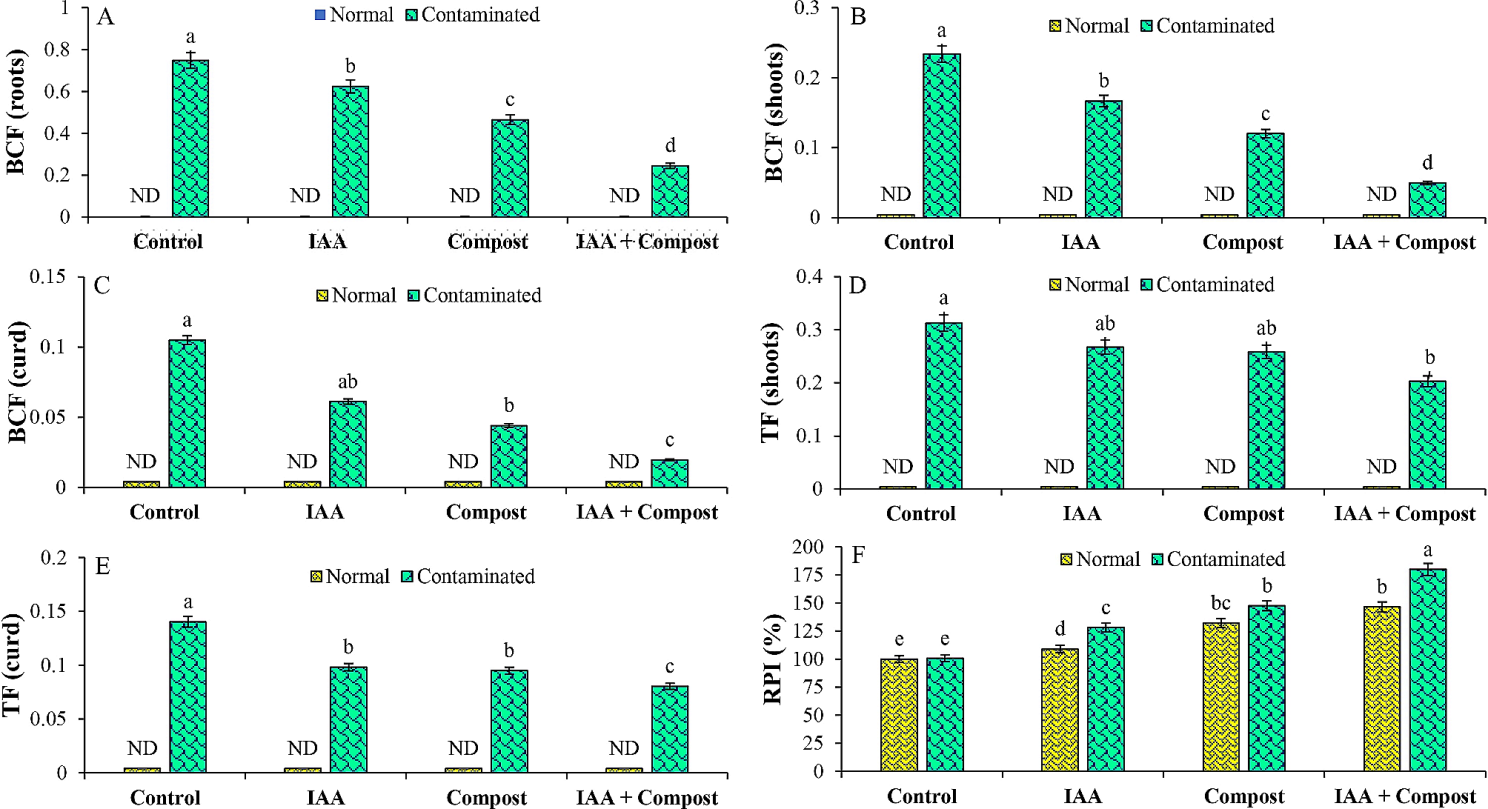
Effect of individual and combined application of IAA and compost on (**A**) Bioconcentration factor of roots, (**B**) Bioconcentration factor of shoots, (**C**) Bioconcentration factor of curd, (**D**) Translocation factor of shoots, (**E**) Translocation factor of curd and (**F**) Relative production index (RPI; %) under Ni stress. Bars displaying same alphabets are statistically non-significant at *P* < 0.05

In terms of metal uptake by different plant parts such as roots, shoots and curd, various indices such as bioconcentration factors (BCF), translocation factors (TF) and relative production index (RPI) were calculated (Fig. 9), and their values indicated the potential of IAA and compost for phytostabilizing Ni in cauliflower rhizosphere. During sole application of compost and IAA, BCF value for different plant parts i.e., roots (0.46 and 0.62), shoots (0.12 and 0.17) and curd (0.04 and 0.06) of cauliflower were observed, where their RPI percentages were increased by 148 and 128% respectively, as compared to corresponding control treatment. In combined application, values of BCF for roots, shoots and curd were found to be 0.24, 0.05 and 0.02 with corresponding RPI increment (180%) (Fig. 9). In terms of translocation factors, in individual and combined application of IAA and compost, the TF values for shoots (0.27, 0.26 and 0.20) and curd (0.10, 0.09 and 0.08) were found to be less than 1, which reflected the phytostabilization of Ni under IAA and compost supplementation (Fig. 9).

### Pearson correlation

**Fig. 10:**
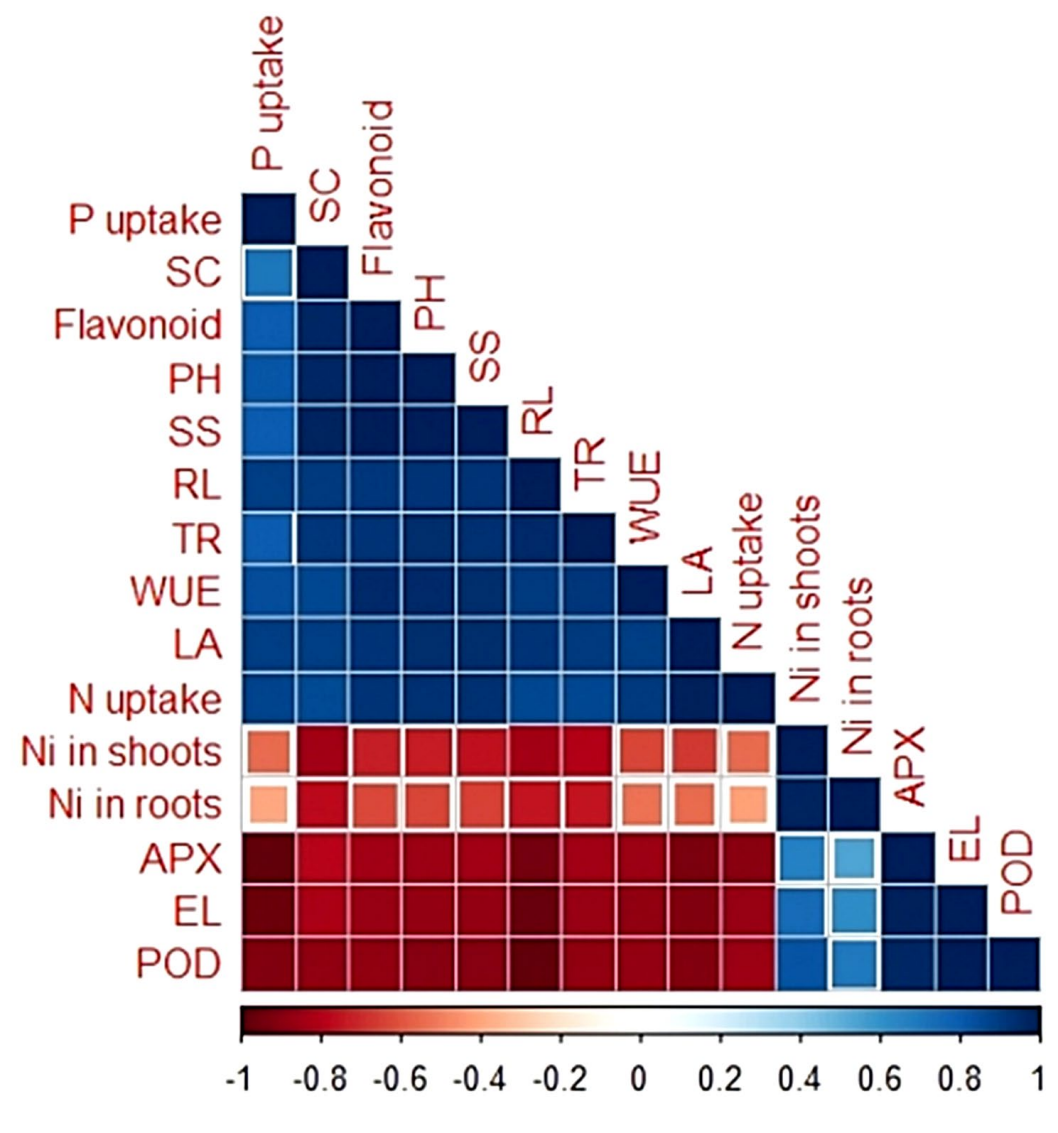
Correlogram between Ni uptake and different plant attributes under Ni stress. Abbreviations: POD = Peroxidase, EL = Electrolyte Leakage, APX = Ascorbate peroxidase, Ni = Nickel, LA = Leaf area, TR = Transpiration rate, WUE = Water use efficiency, N = Nitrogen, RL = Root length, SC = Stomatal conductance, PH = Plant height, SS = Soluble sugars

Pearson correlation describing the interaction between different variables (Fig. 10) was constructed to check the existence of correlation, if any, between Ni uptake and various plant attributes. We observed that Ni uptake in plant shoots was negatively correlated with P uptake (*r* = 0.55), stomatal conductance (*r* = 0.85), plant height (*r* = 0.74), sub-stomatal conductance (*r* = 0.65), root length (*r* = 0.85), transpiration rate (*r* = 0.81), water use efficiency (*r* = 0.64) and leaf area (*r* = 0.67). Furthermore, Ni uptake by plant shoots was positively correlated with antioxidant activities such as APX (*r* = 0.67) and POD (*r* = 0.82) along with EL (*r* = 0.75). Similar negative correlation was pragmatic between Ni uptake in plant roots and other plant attributes (Fig. 10).

## Discussion

Heavy metal (HM) contamination arising from increased industrialization has led to reduced agricultural productivity and deteriorated soil health worldwide. They disrupt normal functions of plants after their entrance to the body leading to their declined productivity and growth. Hence, their remediation by following suitable approaches is imperative to ensure sustained plant growth under contaminated conditions [48]. Despite numerous studies that have reported plant growth inhibition under Ni stress [49], limited research is available regarding combined effect of PGRs and organic amendments in alleviating Ni mediated hazardous effects on plant growth and soil health.

In the present study, we found that application of auxin in association with compost significantly ameliorated the negative effects of Ni stress on cauliflower growth and other attributes. Beneficial effects of auxin under HM contaminated soils have also been reported earlier by Ji et al. [50] and Lin et al. [51] in *Solanum nigrum* L. and *Leersia hexandra* under Cd and Cu contaminations respectively, which might be correlated with auxin induced promotion in plant biomass via enhancement of cell division and growth rates [52]. Similarly, increased plant growth and biomass after compost supplementation are consistent with earlier findings [53]. Beneficial impact of combined application of compost and PGR on plant growth and yield attributes might be due to additive effect of IAA and compost on soil and plant health, leading to improved plant growth as compared to their individual applications. In terms of chlorophyll contents, addition of IAA and compost led to an overall increase in chlorophyll a and b as well as carotenoid contents in leaves, which is in line with previous research findings [54, 55], who suggested that chlorophyll contents in A. *obliquus* and C. *vulgaris* under HM stress can be enhanced by plant growth regulators. The notable increase in chlorophyll content observed in treatments involving IAA, either alone or in combination with compost can be attributed to alterations in the pigment composition of the photosynthetic apparatus. These changes may have resulted in a reduction in the content of light-harvesting chlorophyll proteins (LHCPs), which serves as an adaptive defense mechanism within chloroplasts, leaves, and plants, allowing them to withstand adverse conditions [56]. Consequently, application of compost also minimized the negative impact of Ni on chlorophyll in cauliflower photosynthetic organs, which were also in line with earlier reports [57].

Cauliflower under exposure to Ni-stress exhibited higher production of different antioxidants, which served as defense mechanism employed by plants under HM induced osmotic stress [58]. The SOD functions to scavenge and catalyze the ROS and transforms it into H_2_O_2_ and molecular oxygen under stressed soil environments [59]. This H_2_O_2_ is an intermediate ROS that is toxic to plants. However, it can be converted to water and oxygen by other antioxidant enzymes, such as CAT and POX [60]. It has been well documented that compost offers a crucial role in immobilizing HM, ultimately leading to increased plant growth and antioxidant defense by minimizing the uptake of HMs by plants [53]. Earlier studies have also reported an increase in the antioxidant activities in plants due to application of compost [61]. In this study, combined application resulted in increased antioxidative production than single application. Previous findings supported this result indicating that use of plant growth regulators with organic amendments increased the antioxidant potential of plants in metal-contaminated soil [62]. Our results revealed that treatment of IAA and compost regulated the antioxidant system in Ni-stressed *B. oleracea* plant. Decreased activities of different antioxidative enzymes reduced the MDA contents and alleviated HM stress in plants [63]. Reduced MDA contents under combined application of compost and IAA, as observed in current study, might be correlated with reduced Ni mobility and its subsequent entry to plants.

Moreover, Ni stress exerts deleterious impacts on plants by altering its biochemical attributes leading to increased oxidative stress, and to tackle the situation, plants accumulate intra-cellular osmoprotectants [64]. Reduced productions of soluble sugars, soluble proteins, amino acids, and flavonoid contents were observed in current study, which may be due to suppression of protein synthesis or increased rate of degradation of proteins as observed by [65]. Application of different osmoprotectants such as phenolics, soluble sugars and proteins offer significant roles in imparting tolerance to plants under stressed conditions [66]. Production of these osmoprotectants under stressed conditions indicates their potential to bear the burden of severe environmental stressors by harboring a strong antioxidant response [67]. Increased osmoprotectants after application of compost might be related to composts’ ability to enhance soil fertility owing to their saturation with carbon, hydrogen, and oxygen [68]. Hence, improved soil physicochemical attributes, increased organic matter contents and soil fertility status improved the concentration of osmoprotectants in plants [69]. Concentrations of MDA, and H_2_O_2_ along with electrolyte leakage were significantly decreased under compost treatment, which reflected the tremendous potential of compost to alleviate the Ni mediated osmotic imbalance in plants and its disastrous impacts on cell membranes [67].

In terms of nutrient uptake, Ni stress interfered with their uptake, and ultimately, disrupted their beneficial activities in plant body due to excessive uptake of Ni. Earlier researchers have also reported regarding the reduction in the uptake of essential mineral ions under HM stress [70]. However, application of compost replenished the nutrient balance by reducing the uptake of Ni via inducing chelation, precipitation as well as adsorption of Ni leading to its increased immobilization. Earlier researchers reported regarding the increased uptake of NPK in maize seedlings after treatment with compost [71]. Previous research has shown that the use of organic amendments such as cow manure and compost resulted in the successful stabilization of HMs [72]. Additionally, it has been found that the ability of plants to stabilize HMs can be enhanced by application of plant growth regulators, which improve plant’s growth and physiology under metal stress, thereby increasing its ability for phytostabilization. These findings were reported in a recent study conducted by Zhu et al. [73].

## Conclusion

Nickel (Ni) is a phytotoxic element that exerts adverse impacts on plant growth, physiology, and lessen its yield. It not only interferes with major plant functions but also reduces its overall growth. As a sustainable remediation approach, integrated application of plant growth regulators (PGR) and organic amendment have proved to be effective in immobilizing Ni levels in soil and sustaining plant growth under contaminated conditions. In the current study, we investigated the beneficial role of auxin (IAA) and compost in improving cauliflower growth under Ni contaminated soil conditions. The combined application of auxin and compost mitigated the adverse impacts of Ni stress on cauliflower growth, physiology, yield, and other attributes by suppressing its uptake by plant roots. Phytostabilization of Ni in cauliflower rhizosphere was the key phenomenon behind increased plant growth and yield. To validate these outcomes, further research is needed for understanding the underlying mechanism of this interaction, and to customize the treatment for varying soil types and degrees of Ni contamination.

## Data Availability

All data generated or analyzed during this study are included in this published article.
